# 4-Methyl-2-(2-methyl­anilino)benzoic acid

**DOI:** 10.1107/S2414314623005990

**Published:** 2023-07-14

**Authors:** Chenxin Liu, Sihui Long

**Affiliations:** a School of Chemical Engineering and Pharmacy, Wuhan Institute of Technology, Wuhan, Hubei 430205, People’s Republic of China; Howard University, USA

**Keywords:** crystal structure, twisted conformation, acid–acid dimer

## Abstract

The title compound, C_15_H_15_NO_2_, was obtained by the reaction of 2-chloro-4-methyl-benzoic acid and *o*-toluidine using 2-eth­oxy­ethanol as solvent. Crystals of the title compounds were obtained from crystallization in acetone. The mol­ecule in the crystal is twisted with a dihedral angle between the aromatic rings of 50.86 (5)°. In the crystal structure, the mol­ecules associate to form acid–acid hydrogen-bonded dimers linked by pairwise O—H⋯O hydrogen bonds.

## Abstract 2

Single crystals of 4-methyl-2-(*o*-tolyl­amino)­benzoic acid were obtained from slow evaporation of an acetone solution. The mol­ecule is highly twisted with a dihedral angle between the aromatic rings of 50.86 (5)°. In the crystal structure, the mol­ecules associate to form acid–acid dimers.

## Graphical abstract

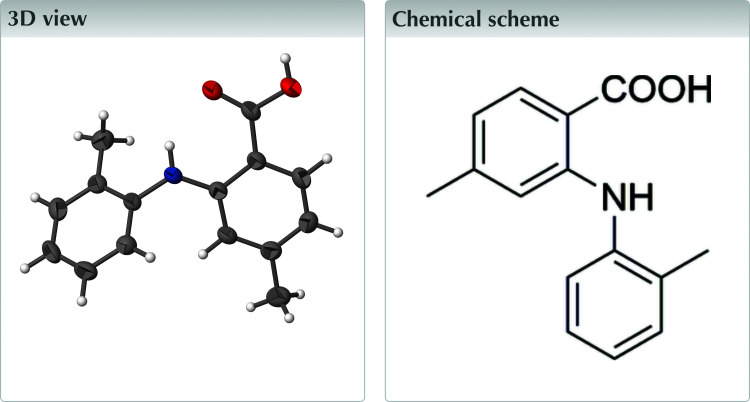

## Structure description

**Figure 1:**
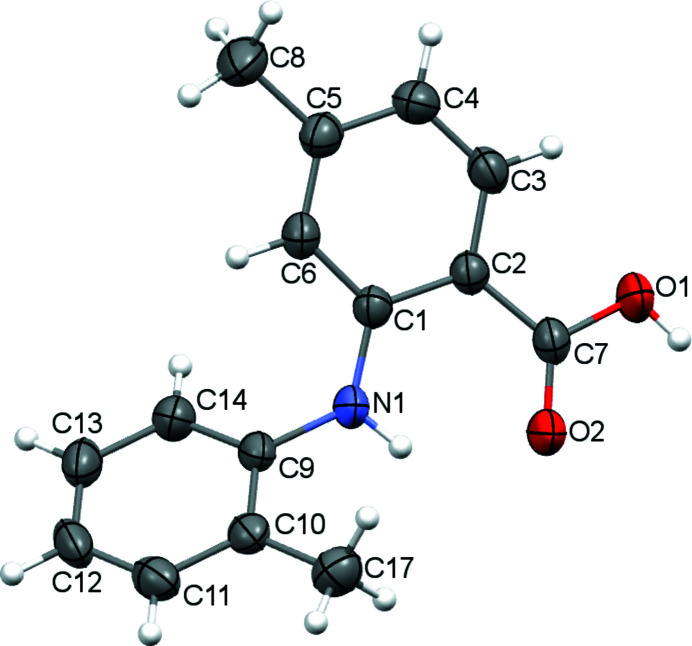
Mol­ecular structure of the title compound, with displacement ellipsoids drawn at the 50% probability level.

**Figure 2:**
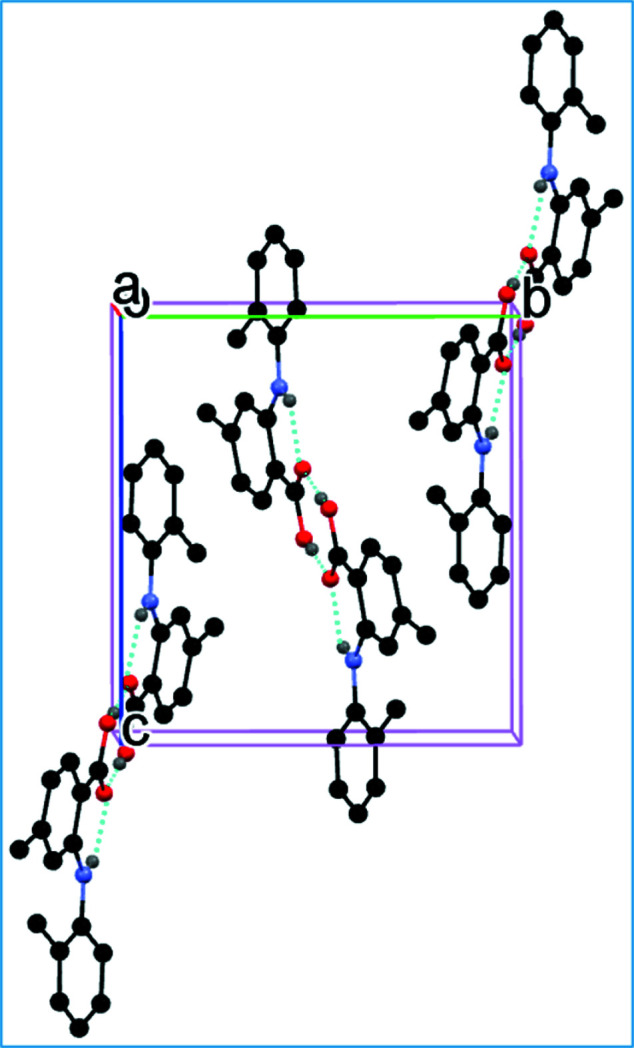
Packing of the mol­ecules in the title compound (for clarity, H atoms not involved in hydrogen bonding are omitted). Hydrogen bonds are indicated by dashed lines.

Anthranilic acids are compounds with great medicinal value. They play an important role in non-steroidal anti-inflammatory (Masubuchi *et al.*, 1998 ▸), anti­bacterial (Abdulkarem *et al.*, 2019 ▸) and anti­viral agents (Inglot 1969 ▸) and other drugs. The title compound has a methyl group on both aromatic rings (Fig. 1 ▸). As a result of steric repulsion, the aromatic rings are not coplanar with a dihedral angle of 50.86 (5)°. In the crystal, two mol­ecules pair up to form a carb­oxy­lic acid–carb­oxy­lic acid hydrogen-bonded dimer. An intra­molecular N1—H1*A*⋯O2 hydrogen bond (Table 1 ▸, Fig. 2 ▸) is also observed.

## Synthesis and crystallization

**Figure 3:**
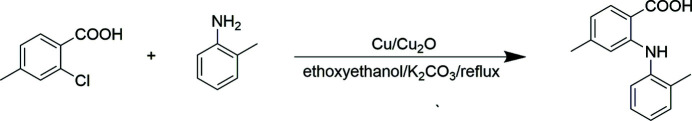
Reaction scheme.

**Figure 4:**
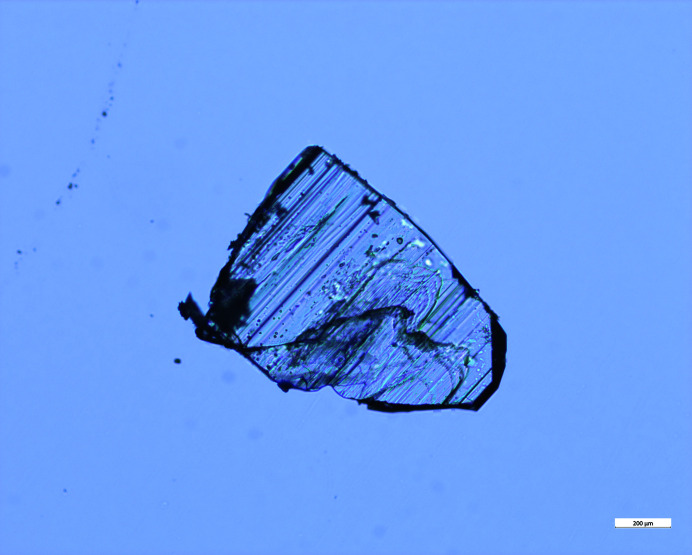
A representative crystal of the title compound.

The title compound was prepared by reacting 2-chloro-4-methyl-benzoic acid and *o*-toluidine in the presence of a catalyst at 403 K (Fig. 3 ▸). The product was purified by column chromatography. Single crystals were obtained by slowly evaporating an acetone solution of the compound (Fig. 4 ▸).

## Refinement

Crystal data, data collection and structure refinement details are summarized in Table 2 ▸.

## Supplementary Material

Crystal structure: contains datablock(s) global, I. DOI: 10.1107/S2414314623005990/bv4047sup1.cif

Structure factors: contains datablock(s) I. DOI: 10.1107/S2414314623005990/bv4047Isup2.hkl

Supporting information file. DOI: 10.1107/S2414314623005990/bv4047Isup3.cml

CCDC reference: 2280189

Additional supporting information:  crystallographic information; 3D view; checkCIF report

## Figures and Tables

**Table 1:** Hydrogen-bond geometry (Å, °)

*D*—H⋯*A*	*D*—H	H⋯*A*	*D*⋯*A*	*D*—H⋯*A*
O1—H1⋯O2^i^	0.82	1.84	2.6570 (17)	174
N1—H1*A*⋯O2	0.86	2.01	2.6942 (17)	136

Symmetry code: (i) 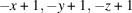 .

**Table 2:** Experimental details

Crystal data	
Chemical formula	C_15_H_15_NO_2_
*M*_r_	241.28
Crystal system, space group	Monoclinic, *P*2_1_/*c*
Temperature (K)	293
*a*, *b*, *c* (Å)	9.6678 (8), 10.9294 (11), 11.7231 (8)
β (°)	93.395 (7)
*V* (Å^3^)	1236.53 (18)
*Z*	4
Radiation type	Cu *K*α
μ (mm^−1^)	0.69
Crystal size (mm)	0.08 × 0.04 × 0.02

Data collection	
Diffractometer	SuperNova, Dual, Cu at zero, Eos
Absorption correction	Multi-scan (*CrysAlis PRO*; Rigaku OD, 2015 ▸)
*T*_min_, *T*_max_	0.919, 1.000
No. of measured, independent and observed [*I* > 2σ(*I*)] reflections	4437, 2285, 1828
*R*_int_	0.019
(sin θ/λ)_max_ (Å^−1^)	0.609

Refinement	
*R*[*F* ^2^ > 2σ(*F* ^2^)], *wR*(*F* ^2^), *S*	0.045, 0.131, 1.04
No. of reflections	2285
No. of parameters	166
H-atom treatment	H-atom parameters constrained
Δρ_max_, Δρ_min_ (e Å^−3^)	0.25, −0.20

Computer programs: *CrysAlis PRO* (Rigaku OD, 2015 ▸), *SHELXS* (Sheldrick, 2008 ▸), *SHELXL* (Sheldrick, 2015 ▸), *OLEX2* (Dolomanov *et al.*, 2009 ▸) and *Mercury* (Macrae *et al.*, 2020 ▸).

## full crystallographic data

### Crystal data

**Table:**

C_15_H_15_NO_2_	*F*(000) = 512
*M**_r_* = 241.28	*D*_x_ = 1.296 Mg m^−^^3^
Monoclinic, *P*2_1_/*c*	Cu *K*α radiation, λ = 1.54184 Å
*a* = 9.6678 (8) Å	Cell parameters from 1387 reflections
*b* = 10.9294 (11) Å	θ = 9.3–69.0°
*c* = 11.7231 (8) Å	µ = 0.69 mm^−^^1^
β = 93.395 (7)°	*T* = 293 K
*V* = 1236.53 (18) Å^3^	Plate, clear light colourless
*Z* = 4	0.08 × 0.04 × 0.02 mm

### Data collection

**Table:**

SuperNova, Dual, Cu at zero, Eos diffractometer	2285 independent reflections
Radiation source: micro-focus sealed X-ray tube, SuperNova (Cu) X-ray Source	1828 reflections with *I* > 2σ(*I*)
Mirror monochromator	*R*_int_ = 0.019
Detector resolution: 16.0733 pixels mm^-1^	θ_max_ = 70.0°, θ_min_ = 4.6°
ω scans	*h* = −11→11
Absorption correction: multi-scan (CrysAlisPro; Rigaku OD, 2015)	*k* = −12→13
*T*_min_ = 0.919, *T*_max_ = 1.000	*l* = −13→10
4437 measured reflections	

### Refinement

**Table:**

Refinement on *F*^2^	Primary atom site location: structure-invariant direct methods
Least-squares matrix: full	Hydrogen site location: inferred from neighbouring sites
*R*[*F*^2^ > 2σ(*F*^2^)] = 0.045	H-atom parameters constrained
*wR*(*F*^2^) = 0.131	*w* = 1/[σ^2^(*F*_o_^2^) + (0.0678*P*)^2^ + 0.2398*P*] where *P* = (*F*_o_^2^ + 2*F*_c_^2^)/3
*S* = 1.04	(Δ/σ)_max_ < 0.001
2285 reflections	Δρ_max_ = 0.25 e Å^−^^3^
166 parameters	Δρ_min_ = −0.20 e Å^−^^3^
0 restraints	

### Special details

**Table:**

Geometry. All esds (except the esd in the dihedral angle between two l.s. planes) are estimated using the full covariance matrix. The cell esds are taken into account individually in the estimation of esds in distances, angles and torsion angles; correlations between esds in cell parameters are only used when they are defined by crystal symmetry. An approximate (isotropic) treatment of cell esds is used for estimating esds involving l.s. planes.
Refinement. The positions of H atoms in N1 and O1 were obtained from the difference Fourier map. Other H atoms were positioned geometrically with C—H = 0.93 for aromatic, and constrained to ride on their parent atoms with *U*_iso_(H) = *xU*_eq_(C,*O*), where *x*=1.5 for all H atoms.

### Fractional atomic coordinates and isotropic or equivalent isotropic displacement parameters (Å^2^)

**Table:**

	*x*	*y*	*z*	*U*_iso_*/*U*_eq_	
O1	0.32010 (13)	0.52719 (14)	0.45925 (9)	0.0527 (4)	
H1	0.396573	0.509295	0.437474	0.079*	
O2	0.44087 (12)	0.53328 (13)	0.62639 (9)	0.0487 (4)	
N1	0.30228 (14)	0.58797 (15)	0.81250 (11)	0.0434 (4)	
H1A	0.377666	0.564178	0.783957	0.052*	
C1	0.19420 (15)	0.61667 (15)	0.73583 (13)	0.0332 (4)	
C2	0.20410 (16)	0.59559 (14)	0.61709 (13)	0.0333 (4)	
C3	0.09126 (17)	0.62616 (17)	0.54234 (13)	0.0409 (4)	
H3	0.096893	0.611557	0.464602	0.049*	
C4	−0.02712 (18)	0.67684 (17)	0.57997 (15)	0.0455 (4)	
H4	−0.100826	0.695348	0.528272	0.055*	
C5	−0.03698 (17)	0.70070 (16)	0.69615 (15)	0.0405 (4)	
C6	0.07217 (16)	0.66986 (16)	0.77176 (13)	0.0375 (4)	
H6	0.064581	0.684862	0.849231	0.045*	
C7	0.33051 (17)	0.54979 (15)	0.57000 (13)	0.0365 (4)	
C8	−0.1651 (2)	0.7593 (2)	0.73824 (18)	0.0614 (6)	
H8A	−0.238018	0.699747	0.739025	0.092*	
H8B	−0.193583	0.825546	0.688383	0.092*	
H8C	−0.145628	0.790187	0.814164	0.092*	
C9	0.30353 (16)	0.59315 (16)	0.93298 (13)	0.0357 (4)	
C10	0.41723 (16)	0.64529 (16)	0.99389 (13)	0.0370 (4)	
C11	0.41862 (18)	0.64593 (17)	1.11262 (14)	0.0452 (4)	
H11	0.494677	0.678781	1.154221	0.054*	
C12	0.3104 (2)	0.59922 (19)	1.17011 (14)	0.0492 (5)	
H12	0.312562	0.602599	1.249460	0.059*	
C13	0.19886 (19)	0.54745 (18)	1.10961 (15)	0.0475 (5)	
H13	0.125187	0.516229	1.148058	0.057*	
C14	0.19640 (18)	0.54187 (17)	0.99152 (14)	0.0426 (4)	
H14	0.122853	0.503693	0.951043	0.051*	
C17	0.53483 (18)	0.69949 (18)	0.93333 (16)	0.0495 (5)	
H17A	0.582818	0.635765	0.895481	0.074*	
H17B	0.597691	0.739472	0.987773	0.074*	
H17C	0.499370	0.757976	0.877931	0.074*	

### Atomic displacement parameters (Å^2^)

**Table:**

	*U*^11^	*U*^22^	*U*^33^	*U*^12^	*U*^13^	*U*^23^
O1	0.0452 (7)	0.0860 (11)	0.0276 (6)	0.0073 (7)	0.0069 (5)	−0.0103 (6)
O2	0.0421 (7)	0.0745 (9)	0.0299 (6)	0.0147 (6)	0.0058 (5)	−0.0046 (6)
N1	0.0337 (7)	0.0716 (11)	0.0253 (7)	0.0098 (7)	0.0055 (5)	−0.0038 (6)
C1	0.0326 (8)	0.0379 (8)	0.0294 (8)	−0.0023 (6)	0.0041 (6)	0.0010 (6)
C2	0.0350 (8)	0.0363 (8)	0.0288 (8)	−0.0023 (6)	0.0046 (6)	−0.0002 (6)
C3	0.0455 (9)	0.0493 (10)	0.0279 (8)	−0.0026 (8)	0.0019 (7)	0.0000 (7)
C4	0.0381 (9)	0.0576 (11)	0.0403 (9)	0.0025 (8)	−0.0021 (7)	0.0065 (8)
C5	0.0374 (9)	0.0438 (9)	0.0407 (9)	0.0020 (7)	0.0076 (7)	0.0061 (7)
C6	0.0388 (9)	0.0450 (9)	0.0295 (8)	0.0025 (7)	0.0082 (6)	0.0001 (7)
C7	0.0419 (9)	0.0422 (9)	0.0260 (7)	−0.0010 (7)	0.0066 (6)	−0.0007 (6)
C8	0.0493 (11)	0.0798 (15)	0.0563 (12)	0.0212 (11)	0.0120 (9)	0.0121 (11)
C9	0.0347 (8)	0.0457 (9)	0.0269 (7)	0.0108 (7)	0.0038 (6)	−0.0002 (7)
C10	0.0364 (8)	0.0400 (9)	0.0347 (8)	0.0094 (7)	0.0029 (6)	−0.0011 (7)
C11	0.0490 (10)	0.0497 (10)	0.0361 (9)	0.0095 (8)	−0.0051 (7)	−0.0046 (8)
C12	0.0610 (11)	0.0618 (12)	0.0251 (8)	0.0175 (9)	0.0044 (8)	0.0031 (8)
C13	0.0463 (10)	0.0587 (11)	0.0390 (9)	0.0109 (9)	0.0151 (8)	0.0124 (8)
C14	0.0363 (8)	0.0560 (11)	0.0358 (9)	0.0029 (8)	0.0046 (7)	0.0026 (8)
C17	0.0411 (9)	0.0543 (11)	0.0531 (11)	−0.0015 (8)	0.0036 (8)	0.0009 (9)

### Geometric parameters (Å, º)

**Table:**

O1—C7	1.3196 (18)	C5—C6	1.379 (2)
O2—C7	1.235 (2)	C5—C8	1.504 (2)
N1—C1	1.374 (2)	C9—C10	1.396 (2)
N1—C9	1.4129 (19)	C9—C14	1.394 (2)
C1—C2	1.420 (2)	C10—C11	1.391 (2)
C1—C6	1.402 (2)	C10—C17	1.498 (2)
C2—C3	1.399 (2)	C11—C12	1.376 (3)
C2—C7	1.459 (2)	C12—C13	1.377 (3)
C3—C4	1.368 (2)	C13—C14	1.385 (2)
C4—C5	1.396 (2)		
			
C1—N1—C9	127.31 (13)	O1—C7—C2	114.81 (14)
N1—C1—C2	120.78 (14)	O2—C7—O1	120.84 (14)
N1—C1—C6	121.27 (14)	O2—C7—C2	124.35 (14)
C6—C1—C2	117.94 (14)	C10—C9—N1	119.17 (14)
C1—C2—C7	122.22 (14)	C14—C9—N1	120.90 (15)
C3—C2—C1	118.69 (14)	C14—C9—C10	119.86 (15)
C3—C2—C7	118.99 (14)	C9—C10—C17	121.04 (15)
C4—C3—C2	122.08 (15)	C11—C10—C9	118.32 (15)
C3—C4—C5	119.82 (15)	C11—C10—C17	120.64 (16)
C4—C5—C8	120.33 (16)	C12—C11—C10	121.71 (17)
C6—C5—C4	119.19 (15)	C11—C12—C13	119.69 (16)
C6—C5—C8	120.47 (16)	C12—C13—C14	119.98 (16)
C5—C6—C1	122.24 (15)	C13—C14—C9	120.36 (17)

### Hydrogen-bond geometry (Å, º)

**Table:**

*D*—H···*A*	*D*—H	H···*A*	*D*···*A*	*D*—H···*A*
O1—H1···O2^i^	0.82	1.84	2.6570 (17)	174
N1—H1*A*···O2	0.86	2.01	2.6942 (17)	136

Symmetry code: (i) −*x*+1, −*y*+1, −*z*+1.
